# Experimental and numerical analysis of the effects of thermal degradation on carbon monoxide oxidation characteristics of a three-way catalyst

**DOI:** 10.1016/j.heliyon.2024.e26592

**Published:** 2024-02-20

**Authors:** Sota Aoyama, Yunosuke Kubo, Ratnak Sok, Jin Kusaka

**Affiliations:** Propulsion and Energy Systems Laboratory, Waseda University, 3-4-1 Okubo, Shinjuku, Tokyo, 169-8555, Japan

**Keywords:** Gasoline engines, Three-way catalyst, Catalyst aging, Thermal degradation, Emissions control, Modeling and simulation

## Abstract

This work investigates oxygen-storage capacity (OSC) changes during thermal degradation in modern three-way catalysts. Two experiments are performed using catalysts with different degradation degrees to evaluate OSC and reaction rates. The CO_2_ production test, where CO and O_2_ are supplied at a constant temperature, shows decreased CO_2_ production with more degraded catalysts and reduced purification. The CO_2_ production test is conducted using transient temperature increases, showing that the maximum CO_2_ production temperature increases with catalyst degradation. The results reveal an increase in activation energy in the oxygen desorption reaction caused by thermal degradation progresses and a decrease in OSC, resulting in temperature increases in the oxygen storage reaction. In the surface reaction and mass transport model considering the 30 elementary reactions, the predicted results are well-validated for CO_2_ production, enabling good oxygen storage predictions based on actual data. These results can be used to predict OSC by formulating the changes in active site density and activation energy due to degradation.

## 1 Introduction

As many nations have been moving toward a carbon-neutral society, spark-ignited (S.I) engines using conventional gasoline and renewable fuels (including e-fuel) equipped with hybridized powertrains are still a fast solution for rapid decarbonization [1] because full battery electric vehicle market introductions still face challenges such as high-battery cost, lack of charging infrastructure, and consumer acceptance. Research and development of efficient gasoline engines and their three-way catalyst (TWC) exhaust after-treatment system (ATS) remain essential. TWCs can reduce the concentrations of carbon monoxide (CO), nitrogen oxides (NOx), and hydrocarbons (HC), and are critical for regulating emissions from vehicles powered by S.I. engines. Advanced gasoline S.I engines are operated under super-lean mixture [2,3], dilution condition [4], strong tumble, high ignition energy to improve brake thermal efficiency [5,6]. Therefore, low exhaust temperature reduces the TWC purification performance. In next-generation gasoline engines such as SPCCI Mazda SKYACTIV-X [7], a high compression ratio (CR = 17) technology is adopted to boost the engine efficiency. Then, a high gasoline direct injection pressure (P = 120 MPa) is utilized to enhance in-cylinder turbulence, flame speed, and spray-jet velocity to avoid irregular combustion under high loads. Such high load, high CR, ultra-high injection pressure may exhibit high exhaust gas temperature, thermally degrading TWC performances. As a result, it is a challenge to develop an innovative TWC-ATS to comply with stringent emission regulations such as US2027, Euro 7, and real driving emissions (RDE) tests. Such an innovative TWC development requires a fast model-based development (MBD) approach to understand thermal performance degradation. A TWC system contains a monolithic substrate coated with precious group metals or PGM (Palladium Pd, Platinum Pt, and Rhodium Rh). Each precious metal has its role in purification performance characteristics [8,9]. However, the price of PGMs, especially Pd (the most commonly supported metal of PGMs) has risen sharply in recent years. Therefore, reducing the usage of PGMs and catalyst development costs is essential. The PGM amounts in TWCs vary among manufacturers, and the characteristics of each precious metal, oxygen storage capacity (OSC) of PGMs, and their degradation must be investigated. Such investigations enable manufacturers and researchers to reduce the amount of PGMs. Therefore, reducing the Pd amount in TWCs is critical in lowering the TWC development cost. TWC purification performances reduce when loaded Pd is lowered, prompting further investigation into the performance degradation.

To improve the purification performance with lowered Pd, ceria-zirconia (CeO_2_–ZrO_2_, CZ) is used as a co-catalyst to enhance OSC [[10], [11], [12]]. However, TWCs are exposed to high exhaust gas temperature under high engine load operation, which eventually causes thermal degradation and purification performance. A methodology to reduce high exhaust gas temperature in high engine load regions is to enrich the air-fuel ratio, lowering the exhaust temperature and thermal degradation. Such air-fuel ratio enrichment inevitably increases the engine fuel consumption, and the TWC purification performance may deteriorate, especially under drive-cycle conditions. Therefore, a further understanding of the TWC thermal degradation, including oxygen storage, is vital for reducing engine fuel consumption and exhaust gas emissions. Recent literature studies have shown that TWC thermal degradation causes a sintering effect and reduces the purification performance due to a reduction of the specific surface area of precious metals [[13], [14], [15]]. The CO reaction rate results [14] suggest that the reduced OSC performance is related to the Pd/CZ interface. As a result, the reduced specific surface area (a quantitative factor) and reaction rate constant (a qualitative factor in the noble metals and OSCs reactions) affect the overall performance of TWCs [15]. Although it is widely known that the surface contact between the precious metals and OSC is lost during the sintering effect, the degradation state varies from catalyst to catalyst, and the presence of the Pd/CZ interface is significant because a large amount of oxygen is absorbed where the precious metals are loaded [15,16]. Several studies reported that the sintering and interface characteristics may differ depending on the supported precious metal [17,18]. However, these studies consider only Pd/PdO redox reactions and utilize different PGM materials, and their experiments were performed with an insufficient oxygen absorption rate.

In addition to the sintering effect, Gong et al. [15] modeled dynamic changes in OSC. They reported that OSC reacts with each gas species independently and further integrated the three-phase boundary (TPB) and OSC with precious metal at S1 reactions. However, since the TWC purification performance has the sintering effect and OSC is the qualitative and quantitative degradation factor, developing a generic TWC degradation prediction model is challenging if all parameters are changed simultaneously. In addition, the reaction schemes are enormous if all reactions of each chemical species in OSC are considered. Rink et al. [19] proposed a reduced dynamic model accounting for thermodynamic data and equilibrium-based oxygen storage models for commercial catalysts to simplify the model. Their model could accurately reproduce experimental values, but they only considered chemical species such as H_2_O, O_2_, and H_2_, ignoring O_2_, NO, CO, and C_3_H_6_ chemical species. Di Maio et al. [20] proposed a similar TWC model to predict CNG engine emissions and reported a high prediction accuracy for CO oxidation reactions. This is likely because CO oxidation reactions occur regardless of the Pd oxidation state [21]. Unfortunately, their model was accurate only under lean and stoichiometric mixture operations. Additionally, although PdO was considered, their model did not account for the adsorption phenomena to each active site and oxygen exchange between OSC and TPB. In actual transient engine operations with frequent lean/rich perturbation, the PdO changes significantly due to oxygen absorption in CZ [22]. Therefore, OSC modeling should be considered early in model development. In previous works of the authors [11,23], such limitations were considered. However, the model accuracy was not optimal because only the particle size changes due to thermal degradation were considered.

The limitations of the previous studies [[15], [16], [17], [18], [19], [20]] are addressed in this work using mini-reactor experiments, and the TWC model accounting for direct reactions with different inlet gases (O_2_, NO, CO, C_3_H_6_) and O_2_ moving in OSC is developed. Although many modeling efforts from the literature focused on the CO oxidation reactions and OSC, the TWC purification performance characteristics during thermal degradation, exhibiting many elementary reactions, including PGMs and OSC, are not well reported. Moreover, the relationship between thermal degradation and CO purification has not been well understood in the reported studies. This present work is extended from the author's previous study [23] by further improving the modeling accuracy of OSC. Moreover, this work considers the OSC reaction rate on fresh and moderately degraded catalysts, and multiple catalyst samples are utilized to improve a more generic model. Multiple reactions (CO, NO, THC) and each characteristic can be predicted accurately without utilizing many reaction schemes [24] at OSC via experiments. The present model is validated with experimental data using fresh, moderately, and severely degraded catalysts to study the oxygen storage characteristics, oxygen storage/release, and CO oxidation reactions occurring on the surface of the storage material and catalyst precious metals. The quantitative and qualitative factors of OSC are considered in the model, including degradation performance, reaction rate parameters, and specific surface area. The development of an elementary reaction mechanism to reproduce CO oxidation characteristics with OSC is reported, considering the reactions in three-way catalysts and during degradation.

## 2 Research methodologies

**Fig. 1:**
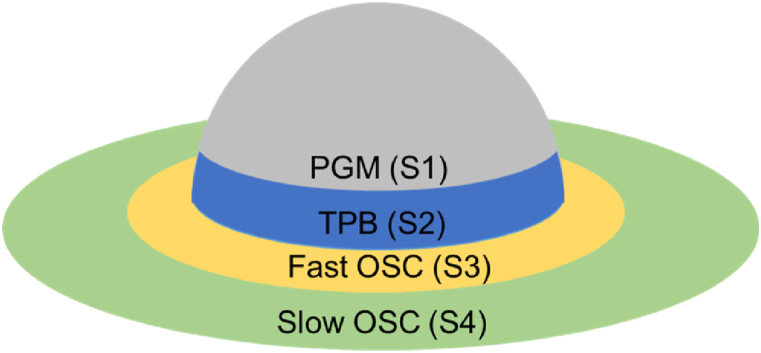
Schematic diagram of TWC on one of the precious metals supported.

Fig. 1 shows the arrangement of active sites in the TWC used in this work, which assumes that the first active site is the PGM surface. The second is the TPB, which exists near the boundary of the PGM and oxygen storage materials. The third is “Fast OSC,” which is responsible for rapidly capturing and releasing oxygen atoms. The fourth is “Slow OSC”, representing a less active site due to its distance from the TPB, and its OSC is less than the Fast OSC. In Fig. 1, S1, S2, S3, and S4 represent PGM, TPB, Fast OSC, and Slow OSC, respectively. Table 1, Table 2, Table 3 list reactions in PGM, TPB, and OSC considered in this work. As shown in Table 1, Table 2, the reaction rate parameters associated with S1 and S2 are obtained experimentally in Ref. [11], and those marked with *were fitted numerically according to the experimental results. Also, R1, R3, R5, R15, R17, R19, R29, and R30 are mainly related to the CO oxidation reaction focused on this research. The authors also assumed that O_2_ is dissociated and adsorbed as O atoms on surfaces PGM (S1) and TPB (S2) as described in R3 and R17 reactions. CO is also adsorbed on PGM (S1) and TPB (S2) surfaces, expressed as R1 and R15, and reacts with O atoms in R5 and R19. One of the PGM (S1) roles and TPB (S2) is to act as an interface between the gas and the solid surface for O_2_ and CO. Excess O atoms on the surface of TPB (S2) move to the Fast OSC site (S3) and are stored. O atoms also transfer to the Slow OSC site (S4) from Fast OSC (S3), as indicated by R29 and R30 in Table 3. Conversely, O atoms can also transfer from Slow OSC (S4) to Fast OSC (S3), and from Fast OSC (S3) to TPB (S2). In other words, for S3 and S4, the reaction model assumes that O_2_ is only supplied through S2. However, further improvements are needed in this respect, including the identification of reactions and reaction rates in OSC.

**Table 1:** Reaction schemes in adsorption, desorption, and surface reaction on PGM active site.

No.	Reactants		Products	Reference
R1	CO + S1	⇄	CO(S1)	Ref [11]
R2	C_3_H_6_ + S1	⇄	C_3_H_6_(S1)	Ref [11]
R3	O_2_ + 2S1	⇄	2O(S1)	Ref [11]
R4	NO + S1	⇄	NO(S1)	Ref [11]
R5	CO(S1) + O(S1)	→	CO_2_ + 2S1	Ref [11]
R6	C_3_H_6_(S1) + 9O(S1)	→	3CO_2_ + 3H_2_O + 10S1	Ref [11]
R7	C_3_H_6_(S1) + 2O(S1)	→	2CH_2_(S1) + CO(S1) + H_2_O	Ref [11]
R8	CH_2_(S1) + 3O(S1)	→	CO_2_ + H_2_O + 4S1	∗
R9	CO(S1) + NO(S1)	→	CO_2_ + N(S1) + S1	Ref [11]
R10	C_3_H_6_(S1) + 9NO(S1)	→	3CO_2_ + 3H_2_O + 9 N(S1) + S1	Ref [11]
R11	CH_2_(S1) + 3NO(S1)	→	CO_2_ + H_2_O + 3 N(S1) + S1	∗
R12	NO(S1) + S1	→	N(S1) + O(S1)	∗
R13	N(S1) + N(S1)	→	N_2_ + 2S1	∗
R14	NO(S1) + N(S1)	→	N_2_O + 2S1	∗

**Table 2:** Reaction schemes in adsorption, desorption and TPB reaction on TPB active site.

No.	Reactants		Products	Reference
R15	CO + S2	⇄	CO(S2)	Ref [11]
R16	C_3_H_6_ + S2	⇄	C_3_H_6_(S2)	Ref [11]
R17	O_2_ + 2S2	⇄	2O(S2)	Ref [11]
R18	NO + S2	⇄	NO(S2)	Ref [11]
R19	CO(S2) + O(S2)	→	CO_2_ + 2S2	Ref [11]
R20	C_3_H_6_(S2) + 9O(S2)	→	3CO_2_ + 3H_2_O + 10S2	Ref [11]
R21	C_3_H_6_(S2) + 2O(S2)	→	2CH_2_(S2) + CO(S2) + H_2_O	Ref [11]
R22	CH_2_(S2) + 3O(S2)	→	CO_2_ + H_2_O + 4S2	∗
R23	CO(S2) + NO(S2)	→	CO_2_ + N(S2) + S2	Ref [11]
R24	C_3_H_6_(S2) + 9NO(S2)	→	3CO_2_ + 3H_2_O + 9 N(S2) + S2	Ref [11]
R25	CH_2_(S2) + 3NO(S2)	→	CO_2_ + H_2_O + 3 N(S2) + S2	∗
R26	NO(S2) + S2	→	N(S2) + O(S2)	∗
R27	N(S2) + N(S2)	→	N_2_ + 2S2	∗
R28	NO(S2) + N(S2)	→	N_2_O + 2S2	∗

**Table 3:** Reaction schemes in oxygen storage and release reaction on OSC active site.

No.	Reactants		Products	Reference
R29	O(S2) + S3	⇄	O(S3) + S2	In this study
R30	O(S3) + S4	⇄	O(S4) + S3	In this study

The active site densities of S1 and S2, shown in Table 4, were estimated as follows.

**Table 4:** Active site density for each catalyst used in the validation.

	Particle size nm	PGM σ(S1) mol/m^3^	TPB σ(S2) mol/m^3^
Fresh CZ	26.1	0	0.8412
Mild	92.8	0.1699	0.0663
Severe	190.7	0.0992	0.0157

First, PGM is assumed to be a hemispherical Pd particle, and TPB is the active site at the interface between the Pd particle and the CZ. The particle sizes of the Fresh CZ, Mild, and Severe catalysts of the PGMs are known by measurement, and the PGM loading is known. The active site density $\sigma_{S1+S2}\left[mol/m^{3}\right]$ (PGM and TPB) for each catalyst was calculated based on the amount of CO adsorbed by the CO adsorption method as shown in Eq (1), in which $x_{CO_{ad}}\left[L/g-PGM\right]$ is the amount of CO adsorption per 1 g of PGM. $y_{metal}\left[g/L\right]$ is precious metal loadings. $y_{all}\left[g/L\right]$ is total loading. $\rho_{cat}\left[kg/m^{3}\right]$ is the catalyst density.

(1) $$\begin{array}{c} \sigma_{S1+S2}=\frac{x_{CO_{ad}}\times y_{metal}}{y_{all}\times 22．4}\times \rho_{cat} \end{array}$$

**Fig. 2:**
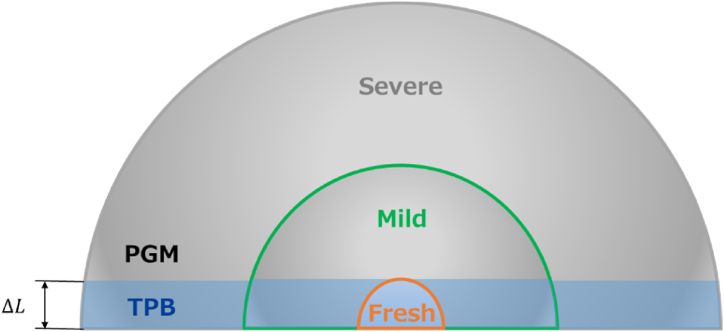
Comparative images of PGM and TPB sites with particle size changes due to degradation.

**Fig. 3:**
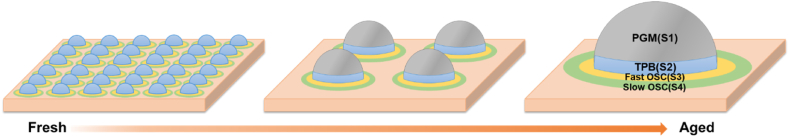
Imaged figure of catalyst degradation.

Fig. 2, Fig. 3 represent the thermal degradation of the catalyst. These figures show that the number of Pd particles per unit area decreases and the particle size increases as sintering degradation progresses. The particle size of each catalyst was determined by measurement, as shown in Table 4, and the diameter of each catalyst was assumed to be uniform. TPB is originally considered to be the intersection of the plane and the spherical surface. However, in the simulations, it must be defined as a surface, and the TPB is assumed to be a band with a constant width ΔL.

Fresh CZ catalyst have smaller precious metal particle sizes, and their radii are smaller than ΔL. That is, the surface was assumed to be completely covered with TPB. For the Fresh CZ catalyst, the active site density $\sigma_{S2_{0}}\left(=\sigma_{S1+S2,0}\right)$ for TPB(S2) is equal to the value calculated in equation (1), thus $\sigma_{S1}$ is zero.

The precious metal particle size of Mild and Severe catalysts increases due to sintering degradation, with radii exceeding ΔL. That is, the surface is covered by PGM and TPB. If the particle size and number of particles of Mild catalyst are $d_{M}\left[m\right]$ and $n_{M}\left[1/m^{3}\right]$, respectively, and the particle size and number of Fresh CZ catalyst are $d_{0}\left[m\right]$ and $n_{0}\left[1/m^{3}\right]$, as shown in equation (2).

(2) $$\begin{array}{c} \frac{4}{3}\pi {\left(\frac{d_{0}}{2}\right)}^{3}\times n_{0}=\frac{4}{3}\pi {\left(\frac{d_{M}}{2}\right)}^{3}\times n_{M} \end{array}$$

Therefore, the particle number $n_{M}$ of Mild catalyst can be estimated using equation (3):

(3) $$\begin{array}{c} n_{M}={\left(\frac{d_{0}}{d_{M}}\right)}^{3}n_{0} \end{array}$$

Since the ratio of TPB active site densities of Fresh CZ and Mild catalysts is the ratio of the number of TPB(Pd_b_) between Pd and CZ per unit volume $N_{Pd_{b}}$, equation (4) can be formulated using the active site density $\sigma_{S2_{0}}$ and number of Pd_b_ $N_{Pd_{b},0}$ for Fresh CZ catalyst and $\sigma_{S2,M}$ and number of Pd_b_ $N_{Pd_{b},M}$ for Mild catalyst.

(4) $$\begin{array}{c} \sigma_{S2,M}=\sigma_{S2_{0}}\times \frac{N_{Pd_{b},M}}{N_{Pd_{b},0}} \end{array}$$

The ratio of the number of Pd_b_ can be calculated using the number of Pd_b_ per particle size, $n_{Pd_{b}}\left[mol\right]$, using equation (5) as follows.

(5) $$\begin{array}{c} N_{Pd_{b}}=n\times n_{Pd_{b}} \end{array}$$

Also, the number of Pd_b_ per particle size for Fresh CZ and Mild catalysts is the ratio of the Pd_b_ surface area per particle size. Therefore, the TPB active site density of the Mild catalyst can be calculated as:

(6) $$\begin{array}{c} ∴\sigma_{S2,M}={\left(\frac{d_{0}}{d_{M}}\right)}^{2}\sigma_{S2_{0}} \end{array}$$

$\sigma_{S2_{0}}$ can be calculated from the CO adsorption method described above and the assumption that the entire hemisphere is covered with TPB for the Fresh CZ catalyst. Therefore, $\sigma_{S2,M}$ for the Mild catalyst can also be obtained using Eq (6).

Since the area of the precious metal in the Mild catalyst is the hemisphere of diameter d minus the area of the strip TPB, its density $\sigma_{S1,M}$ is calculated by equation (7).

(7) $$\begin{array}{c} \sigma_{S1,M}=\sigma_{S1+S2,M}-\sigma_{S2,M} \end{array}$$

Similarly, the densities of PGM and TPB for Severe catalysts can be calculated.

The active site densities of S3 and S4 were estimated from the CO_2_ production test and the S3 reaction rate parameters from the CO-TPR test.

**Fig. 4:**
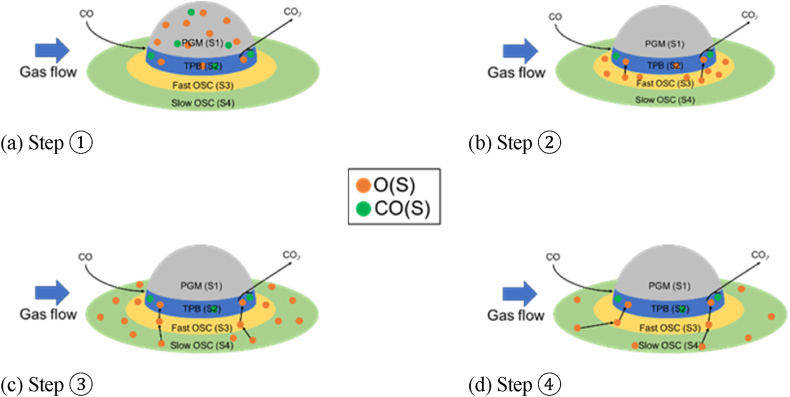
Separation of CO and O_2_ in the CO_2_ production test reaction.

The reaction path considered in this study is shown in Fig. 4 and is explained as follows. First, the O atoms adsorbed on PGM and TPB react as shown in step ① (Fig. 4a) for the reaction in which CO_2_ is produced by adsorption of CO and O_2_ on the catalyst. In step ② (Fig. 4b), when the oxygen adsorption at the TPB decreases, the O atoms in Fast OSC are transferred to TPB, so the purification rate is not reduced. In step ③ (Fig. 4c), when the amount of O in Fast OSC decreases, the O in Slow OSC moves to TPB through Fast OSC and reacts with CO. In step ④ (Fig. 4d), when the amount of oxygen adsorption in Slow OSC is reduced, the purification process falters. Table 5, Table 6, Table 7 list the reaction rate equations used in this work. A is a pre-exponential factor. E is the activation energy. R is the gas constant. T is temperature. $\theta_{i}$ is the coverage ratio of species i. σ is active site density. γ is the coefficient of the i species coverage ratio. The response parameters of the OSC are described in the result section, along with the analysis method.

**Table 5:** Reaction rate equation for adsorption, desorption and surface reaction on PGM active site.

No.		Reaction rate equation
R1	forward	$A_{R1\_f}∙\exp\left(-\frac{E_{a,R1\_f}}{RT}\right)\;X_{CO}\;\theta_{S1}\;\sigma_{1}$
	backward	$A_{R1\_b}∙\exp\left(-\frac{E_{a,R1\_b}\left(1-\gamma \theta_{CO}\right)}{RT}\right)\;\theta_{CO}\;\sigma_{1}$
R2	forward	$A_{R2\_f}∙\exp\left(-\frac{E_{a,R2\_f}}{RT}\right)\;X_{C_{3}C_{6}}\;\theta_{S1}\;\sigma_{1}$
	backward	$A_{R2\_b}∙\exp\left(-\frac{E_{a,R2\_b}\left(1-\gamma \theta_{C_{3}H_{6}}\right)}{RT}\right)\;\theta_{C_{3}H_{6}}\;\sigma_{1}$
R3	forward	$A_{R3\_f}∙\exp\left(-\frac{E_{a,R2\_f}}{RT}\right)\;X_{O_{2}}\;\theta_{S1}^{2}\;\sigma_{1}$
	backward	$A_{R3\_b}∙\exp\left(-\frac{E_{a,R3\_b}\left(1-\gamma \theta_{O}\right)}{RT}\right)\;\theta_{O}^{2}\;\sigma_{1}$
R4	forward	$A_{R4\_f}∙\exp\left(-\frac{E_{a,R4\_f}}{RT}\right)\;X_{NO}\;\theta_{S1}\;\sigma_{1}$
	backward	$A_{R4\_b}∙\exp\left(-\frac{E_{a,R4\_b}\left(1-\gamma \theta_{NO}\right)}{RT}\right)\;\theta_{NO}\;\sigma_{1}$
R5	forward	$A_{R5}∙\exp\left(-\frac{E_{a,R5}\left(1-\gamma \theta_{CO}\right)}{RT}\right)\;\theta_{CO}\;\theta_{O}\;\sigma_{1}$
R6	forward	$A_{R6}∙\exp\left(-\frac{E_{a,R6}\left(1-\gamma \theta_{C_{3}H_{6}}\right)}{RT}\right)\;\theta_{C_{3}H_{6}}\;\theta_{O}^{9}\;\sigma_{1}$
R7	forward	$A_{R7}∙\exp\left(-\frac{E_{a,R7}\left(1-\gamma \theta_{C_{3}H_{6}}\right)}{RT}\right)\;\theta_{C_{3}H_{6}}\;\theta_{O}^{2}\;\sigma_{1}$
R8	forward	$A_{R8}∙\exp\left(-\frac{E_{a,R8}\left(1-\gamma \theta_{CH_{2}}\right)}{RT}\right)\;\theta_{CH_{2}}\;\theta_{O}^{3}\;\sigma_{1}$
R9	forward	$A_{R9}∙\exp\left(-\frac{E_{a,R9}\left(1-\gamma \theta_{CO}\right)}{RT}\right)\;\theta_{CO}\;\theta_{NO}\;\sigma_{1}$
R10	forward	$A_{R10}∙\exp\left(-\frac{E_{a,R10}\left(1-\gamma \theta_{C_{3}H_{6}}\right)}{RT}\right)\;\theta_{C_{3}H_{6}}\;\theta_{NO}^{9}\;\sigma_{1}$
R11	forward	$A_{R11}∙\exp\left(-\frac{E_{a,R11}\left(1-\gamma \theta_{CH_{2}}\right)}{RT}\right)\;\theta_{CH_{2}}\;\theta_{NO}^{3}\;\sigma_{1}$
R12	forward	$A_{R12}∙\exp\left(-\frac{E_{a,R12}\left(1-\gamma \theta_{C_{3}H_{6}}\right)}{RT}\right)\;\theta_{NO}\;\theta_{S1}\;\sigma_{1}$
R13	forward	$A_{R13}∙\exp\left(-\frac{E_{a,R13}\left(1-\gamma \theta_{N}\right)}{RT}\right)\;\theta_{N}^{2}\;\sigma_{1}$
R14	forward	$A_{R14}∙\exp\left(-\frac{E_{a,R14}}{RT}\right)\;\theta_{NO}\;\theta_{N}\;\sigma_{1}$

**Table 6:** Reaction rate equation for adsorption, desorption and TPB reaction on TPB active site.

No.		Reaction rate equation
R15	forward	$A_{R15\_f}∙\exp\left(-\frac{E_{a,R15\_f}}{RT}\right)\;X_{CO}\;\theta_{S2}\;\sigma_{2}$
	backward	$A_{R15\_b}∙\exp\left(-\frac{E_{a,R15\_b}\left(1-\gamma \theta_{CO}\right)}{RT}\right)\;\theta_{CO}\;\sigma_{2}$
R16	forward	$A_{R16\_f}∙\exp\left(-\frac{E_{a,R16\_f}}{RT}\right)\;X_{C_{3}C_{6}}\;\theta_{S2}\;\sigma_{2}$
	backward	$A_{R16\_b}∙\exp\left(-\frac{E_{a,R16\_b}\left(1-\gamma \theta_{C_{3}H_{6}}\right)}{RT}\right)\;\theta_{C_{3}H_{6}}\;\sigma_{2}$
R17	forward	$A_{R17\_f}∙\exp\left(-\frac{E_{a,R17\_f}}{RT}\right)\;X_{O_{2}}\;\theta_{S2}^{2}\;\sigma_{2}$
	backward	$A_{R17\_b}∙\exp\left(-\frac{E_{a,R17\_b}\left(1-\gamma \theta_{O}\right)}{RT}\right)\;\theta_{O}^{2}\;\sigma_{2}$
R18	forward	$A_{R18\_f}∙\exp\left(-\frac{E_{a,R18\_f}}{RT}\right)\;X_{NO}\;\theta_{S2}\;\sigma_{2}$
	backward	$A_{R18\_b}∙\exp\left(-\frac{E_{a,R18\_b}\left(1-\gamma \theta_{NO}\right)}{RT}\right)\;\theta_{NO}\;\sigma_{2}$
R19	forward	$A_{R19}∙\exp\left(-\frac{E_{a,R19}\left(1-\gamma \theta_{CO}\right)}{RT}\right)\;\theta_{CO}\;\theta_{O}\;\sigma_{2}$
R20	forward	$A_{R20}∙\exp\left(-\frac{E_{a,R20}\left(1-\gamma \theta_{C_{3}H_{6}}\right)}{RT}\right)\;\theta_{C_{3}H_{6}}\;\theta_{O}^{9}\;\sigma_{2}$
R21	forward	$A_{R21}∙\exp\left(-\frac{E_{a,R21}\left(1-\gamma \theta_{C_{3}H_{6}}\right)}{RT}\right)\;\theta_{C_{3}H_{6}}\;\theta_{O}^{2}\;\sigma_{2}$
R22	forward	$A_{R22}∙\exp\left(-\frac{E_{a,R22}\left(1-\gamma \theta_{CH_{2}}\right)}{RT}\right)\;\theta_{CH_{2}}\;\theta_{O}^{3}\;\sigma_{2}$
R23	forward	$A_{R23}∙\exp\left(-\frac{E_{a,R23}\left(1-\gamma \theta_{CO}\right)}{RT}\right)\;\theta_{CO}\;\theta_{NO}\;\sigma_{2}$
R24	forward	$A_{R24}∙\exp\left(-\frac{E_{a,R24}\left(1-\gamma \theta_{C_{3}H_{6}}\right)}{RT}\right)\;\theta_{C_{3}H_{6}}\;\theta_{NO}^{9}\;\sigma_{2}$
R25	forward	$A_{R25}∙\exp\left(-\frac{E_{a,R25}\left(1-\gamma \theta_{CH_{2}}\right)}{RT}\right)\;\theta_{CH_{2}}\;\theta_{NO}^{3}\;\sigma_{2}$
R26	forward	$A_{R26}∙\exp\left(-\frac{E_{a,R26}\left(1-\gamma \theta_{C_{3}H_{6}}\right)}{RT}\right)\;\theta_{NO}\;\theta_{S2}\;\sigma_{2}$
R27	forward	$A_{R27}∙\exp\left(-\frac{E_{a,R27}\left(1-\gamma \theta_{N}\right)}{RT}\right)\;\theta_{N}^{2}\;\sigma_{2}$
R28	forward	$A_{R28}∙\exp\left(-\frac{E_{a,R28}}{RT}\right)\;\theta_{NO}\;\theta_{N}\;\sigma_{1}$

**Table 7:** Reaction rate equation for oxygen storage and release reaction on OSC active site.

No.		Reaction rate equation
R29	forward	$A_{R29\_f}∙\exp\left(-\frac{E_{a,R29\_f}\left(1-\gamma \theta_{S3}\right)}{RT}\right)\;\theta_{O\left(S2\right)}\;\theta_{S3}\;\sigma_{S2}\;\sigma_{S3}$
	backward	$A_{R29\_b}∙\exp\left(-\frac{E_{a,R29\_b}(1-\gamma \theta_{O\left(S3\right)}}{RT}\right)\;\theta_{S2}\;\theta_{O\left(S3\right)}\;\sigma_{S2}\;\sigma_{S3}$
R30	forward	$A_{R30\_f}∙\exp\left(-\frac{E_{a,R30\_f}\left(1-\gamma \theta_{S4}\right)}{RT}\right)\;\theta_{O\left(S3\right)}\;\theta_{S4}\;\sigma_{S3}\;\sigma_{S4}$
	backward	$A_{R30\_b}∙\exp\left(-\frac{E_{a,R30_{b}}\left(1-\gamma \theta_{O(S4})\right)}{RT}\right)\;\theta_{S3}\;\theta_{O\left(S4\right)}\;\sigma_{S3}\;\sigma_{S4}$

**Fig. 5:**
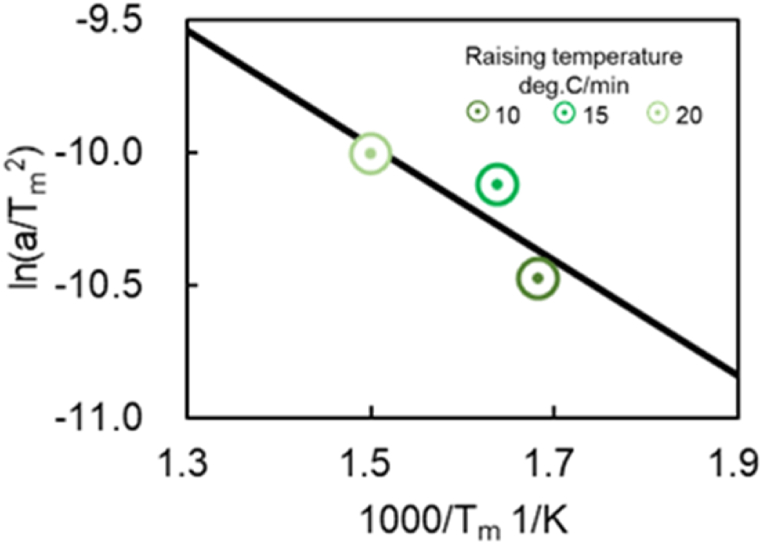
Example of an Ozawa plot applied to the CO-TPR test results conducted in this study.

As mentioned above, the reaction rate parameters associated with S1 and S2 are obtained experimentally in Ref. [11]. However, for model simplification, the previous work did not consider all TWC reactions, including thermal degradation and OSC, and a generic model was not developed. These shortcomings are addressed in the presented study. Two tests were carried out to estimate the activation energy and pre-exponential factor of R29 backward and site densities of S3 and S4. First, this work carried out a CO-TPR (CO-Temperature Programed Reduction) test to find the activation energy and pre-exponential factor of R29 backward. This test measures the CO oxidation reaction behavior with O atoms as indicated by R5 and R19 when the temperature rises at a constant rate. Next, an Ozawa plot [25,26] was used to estimate reaction rate constants. $T_{m}$ is the temperature at which the reaction rate peaks when the temperature is increased at a constant rate. With $T_{m}$, an Ozawa plot shown in Fig. 5 was drawn. The left-hand side of Eq (8) is the vertical axis of Fig. 5 and $T_{m}$ is the horizontal axis. A linear relationship between plots was approximated using the least squares method. Thus, from the slope and intercept of the line, the activation energy and pre-exponential factor can be estimated because the slope of the line is -ΔE/R, and the intercept term includes the pre-exponential factor A.

CO-TPR tests were carried out at various temperature increase rates to provide data for the Ozawa plot for each temperature increase rate. a is rate of temperature increase in [K/min]. $T_{m}$ is the temperature at the peak in [K]. ΔE is the activation energy in [kJ/mol]. A is a pre-exponential factor. R is gas constant in [kJ/(K∙mol)].

(8) $$\begin{array}{c} \ln\frac{a}{T_{m}^{2}}=-\frac{\Delta E}{RT_{m}}+\ln\frac{AR}{\Delta E} \end{array}$$

Secondly, CO_2_ production experiments were performed to find the active site densities of S3 and S4. These experiments were done at steady and high temperatures to confirm O_2_ movement from OSC.

## 3 Experimental method

### 3.1 The experimental setup

**Fig. 6:**
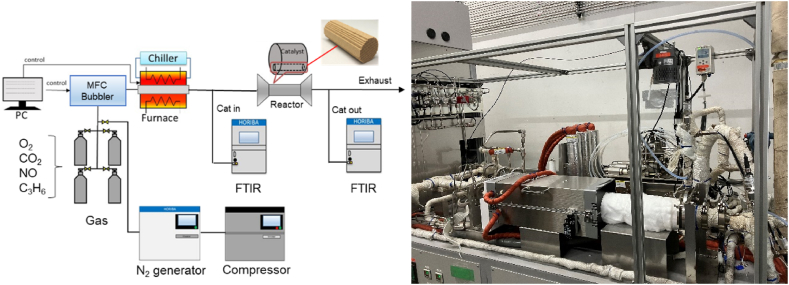
Schematic of the experimental setup used in the reactor test.

Fig. 6 shows a schematic of the experimental setup. The HORIBA SIGU-1000 catalyst testbench has a mass flow controller, a bubbler for water, and a heating furnace and was used to generate simulated exhaust gases with various gas compositions at desired temperature ranges. The exhaust gas is introduced into the catalyst test piece installed in the holder. Gas concentrations upstream and downstream of the catalyst were measured by a HORIBA FTIR gas analyzer (MEXA-ONE-FT).

### 3.2 Catalyst sample specifications

In this work, four full-sized catalysts (Fresh Al_2_O_3_, Fresh CZ, Mild aged, and Severe aged) were cut to form test pieces with diameters and axial lengths of 20 mm and 50 mm, respectively. The catalyst specifications are listed in Table 8, Table 9. Four types of catalysts were used in this study in which Pd was used as the precious metal, mainly ceria-zirconia as the oxygen storage material. Fresh Al_2_O_3_ was the catalyst with Pd supported on Al_2_O_3_ without an OSC function. Fresh CZ, Mild aged (Mild), and Severe aged (Severe) were catalysts of different ages combined with OSC. The temperatures used for endurance treatment were 900 and 1000 °C for Mild and Severe, respectively. The duration of endurance treatment was 40 h for both catalysts. Also, the CO adsorption amount per Pd and dispersion shown in Table 9 are values obtained by the CO adsorption method. This data was measured on catalysts after light-off and mode tests. Pd catalysts supported on Al_2_O_3_ like Fresh Al_2_O_3_ catalysts are large in number of modes of adsorption (bridge) where one CO molecule is adsorbed on two Pd atoms. In addition, the dispersion may be estimated low because the dispersion is evaluated using the CO/Pd = 1 stoichiometry.

**Table 8:** Specifications of a TWC test piece for Fresh Al_2_O_3_ and Fresh CZ, Mild, and Severe catalysts.

Diameter × Length	mm	20 × 50	
PGM loading	g/L	2.0	
PGM active site density	mol/m^3^	1.45	(Fresh Al_2_O_3_)
		8.41 × 10^−1^	(Fresh CZ)
		2.36 × 10^−1^	(Mild)
		1.15 × 10^−1^	(Severe)
Support material		CZ (Ce/Zr/La/Nd = 40/50/4/6) A (3% La–Al_2_O_3_:γ)	
Binder		Al_2_O_3_	
Substrate		Cordierite	
Cell density	cpsi	600	
Wall thickness	mil	4.3	
Cell geometry		Square	
Layering		Mono layer	
Wash-coat loading	g/L	100 (+ Binder 10)

**Table 9:** Specifications obtained by the CO adsorption method for Fresh Al_2_O_3_, Fresh CZ, Mild, and Severe catalysts.

	CO adsorption amount per Pd mL/g-PGM	Dispersion %
Fresh Al_2_O_3_	16.20	7.69
Fresh CZ	9.42	4.48
Mild	2.65	1.26
Severe	1.29	0.61

### 3.3 Experimental procedure

#### 3.3.1 CO-TPR (CO-Temperature Programed Reduction) test

**Fig. 7:**
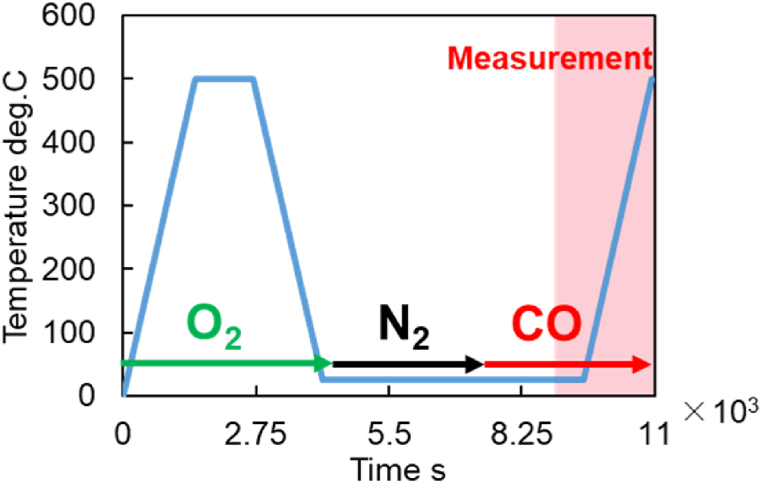
Experimental method of CO-TPR test (time history of introduction timing and temperature for each gas type).

As described earlier, the test investigates the reaction behavior of CO and O_2_ when the temperature is increased at a constant rate. The general flow of this test is shown in Fig. 7. Details of this test procedure are as follows. First, the temperature was raised to 500 °C. Second, OSC fully adsorbed O_2_ by cooling to the temperature at which the measurement started. Third, CO and N_2_ were introduced into the catalyst test piece, and the temperature gradually rose constantly. The CO_2_ production concentration during this was measured, and the experimental conditions are listed in Table 10. The available catalyst heater sets the temperature increase rates to an appropriate range.

**Table 10:** Experiment conditions for the CO-TPR test.

CO	ppm	2000
O_2_	%	5.0
N_2_	%	Balance
Space velocity	h^−1^	5.0 × 10^4^
Temperature	^o^C	25 (room temperature) ∼ 500
Rate of temperature increase	^o^C/min	10，15，20
Time to start of measurement	s	9525

#### 3.3.2 CO_2_ production test

**Fig. 8:**
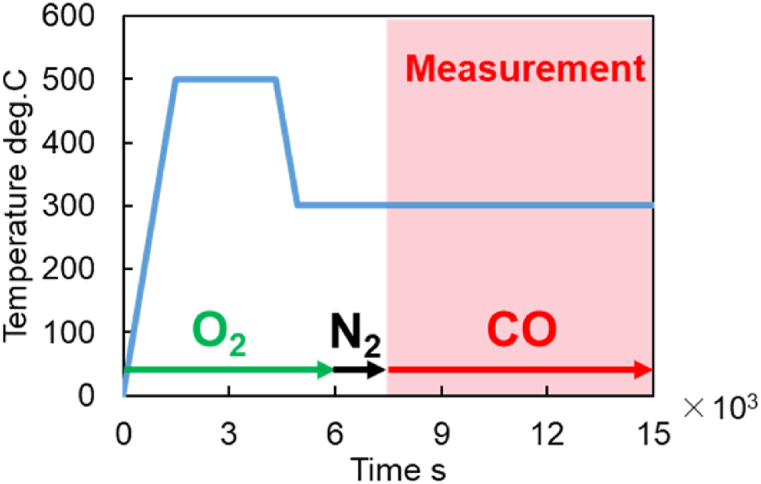
Experimental method of CO_2_ production test (time history of introduction timing and temperature for each gas type).

As described earlier, this test evaluated the behavior of O_2_ moving from OSC to the TPB surface at a steady temperature. The general flow of this test is shown in Fig. 8. Firstly, OSC fully adsorbed O_2_ by raising the temperature to 500 °C and cooling to the starting temperature as for the CO-TPR test. Secondly, CO and N_2_ were only introduced into the gas at a steady temperature. From this point, CO_2_ production concentration was measured. The conditions for this test are shown in Table 11. The measurement temperature was set at a condition at which the OSC functions.

**Table 11:** Experiment conditions for the CO_2_ production test.

CO	ppm	2000
O_2_	ppm	1000
N_2_	%	Balance
Space velocity	h^−1^	5.0 × 10^4^
Temperature	deg.C	300，400
Test time	s	15000

## 4 Experimental results and discussion

### 4.1 CO-TPR test

**Fig. 9:**
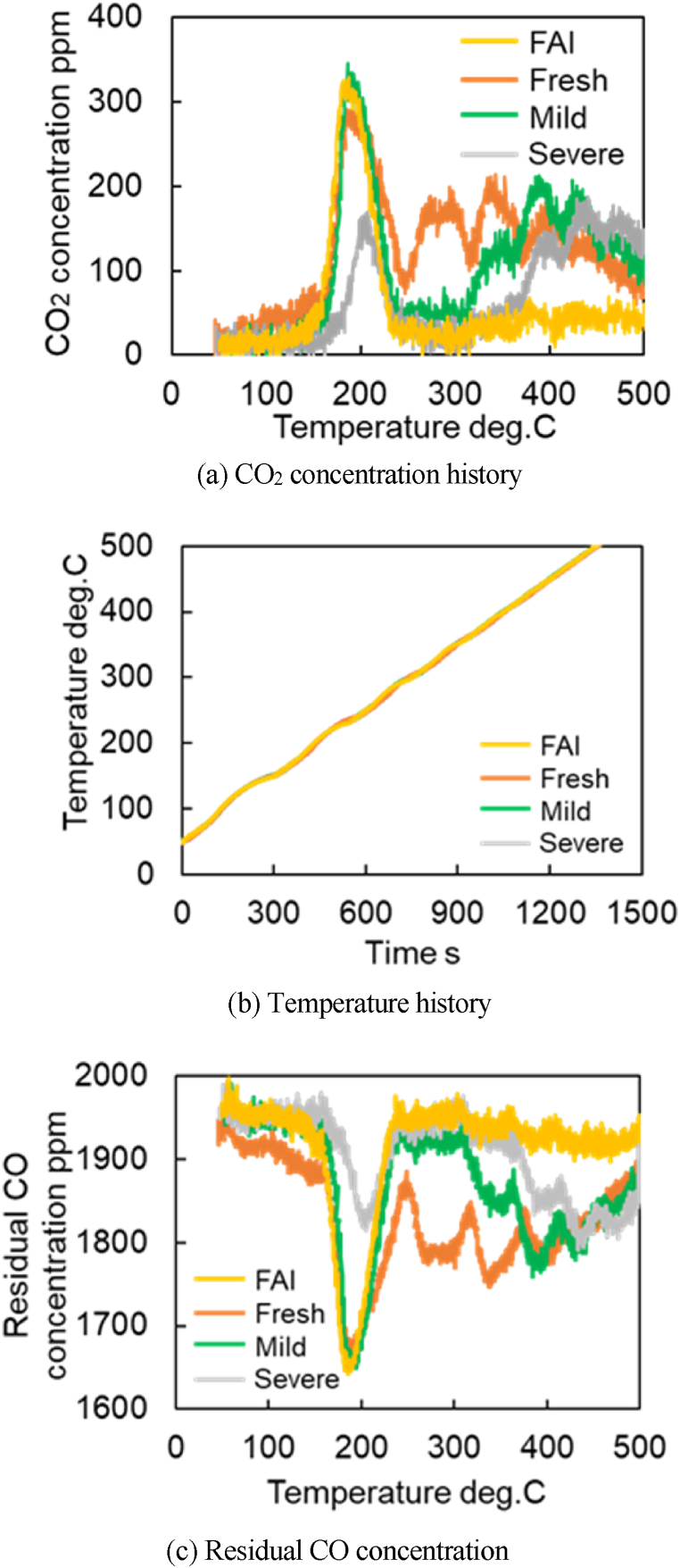
Results of CO-TPR test at 20 deg.C/min.

**Fig. 10:**
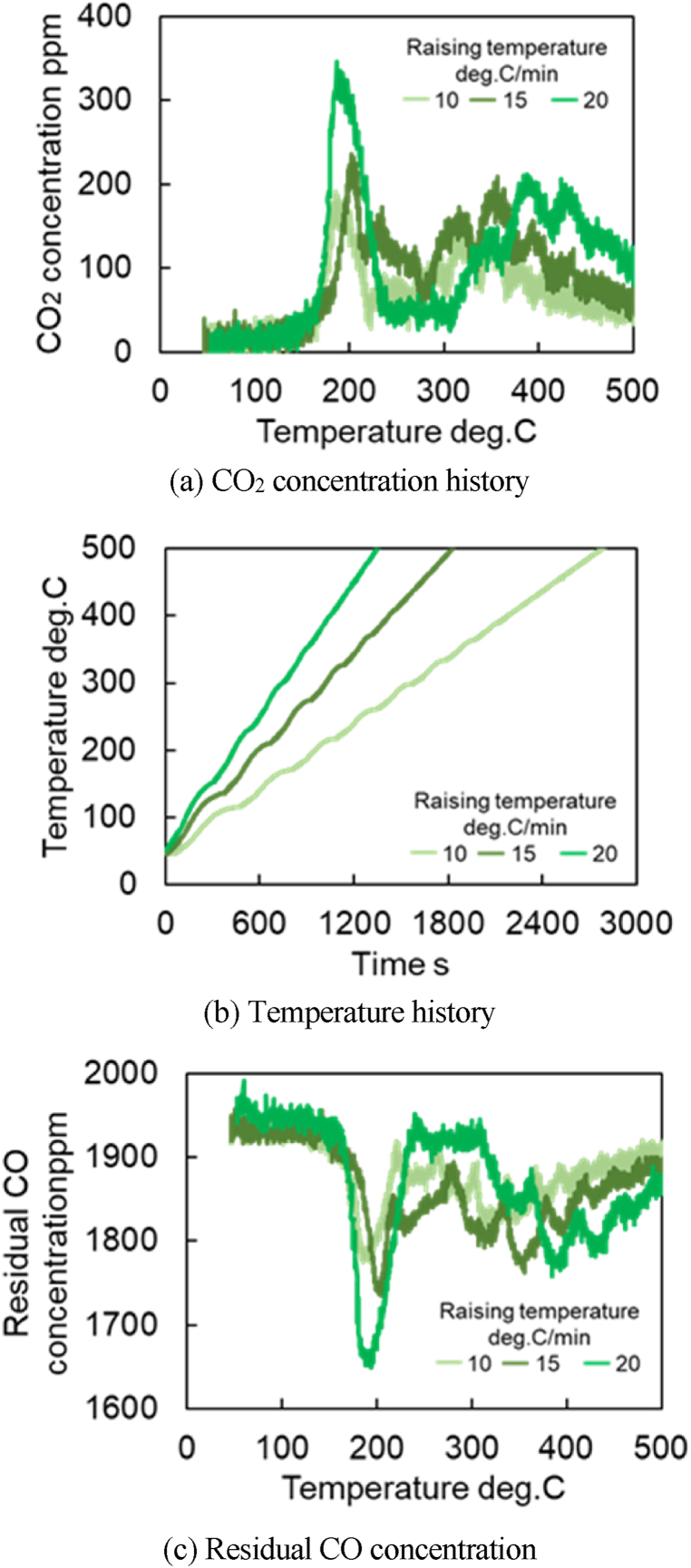
Results of CO-TPR test for Mild catalyst under various temperature increase rates.

Fig. 9a–c shows the amount of CO_2_ produced, the time history of temperature, and the residual CO concentration when the temperature is increased at 20 °C/min, respectively. These results (Fig. 9a–c) show that for three catalysts (Fresh CZ, Mild, Severe), the first peak appears near 200 °C, and the subsequent peaks appear after 250 °C. For Fresh Al_2_O_3_ catalyst, only one peak occurs at 200 °C. The first peaks are strongly influenced by the reactions at the precious metal surfaces, while the reactions due to OSC cause the second and subsequent peaks. Then, the temperature at the second peak of CO_2_ concentration could be extracted, and the reaction rate parameters for R29 were estimated. It is observed in Fig. 9a that the temperature of the second peaks for Mild and Severe catalysts shift to a higher temperature (>350 °C) due to catalyst aging. Fig. 10a shows the CO-TPR test results for the Mild catalyst at different temperature increase rates, while temperature rising rate and residual CO concentration are illustrated in Fig. 10b and c, respectively. Fig. 10a shows that all CO_2_ concentrations of all catalysts oscillate, likely caused by small oscillations in temperature, as shown in Fig. 10b. As a result, the moving average of CO_2_ concentrations was used for extracting the temperature at the second peak of CO_2_ production.

### 4.2 CO_2_ production test

**Fig. 11:**
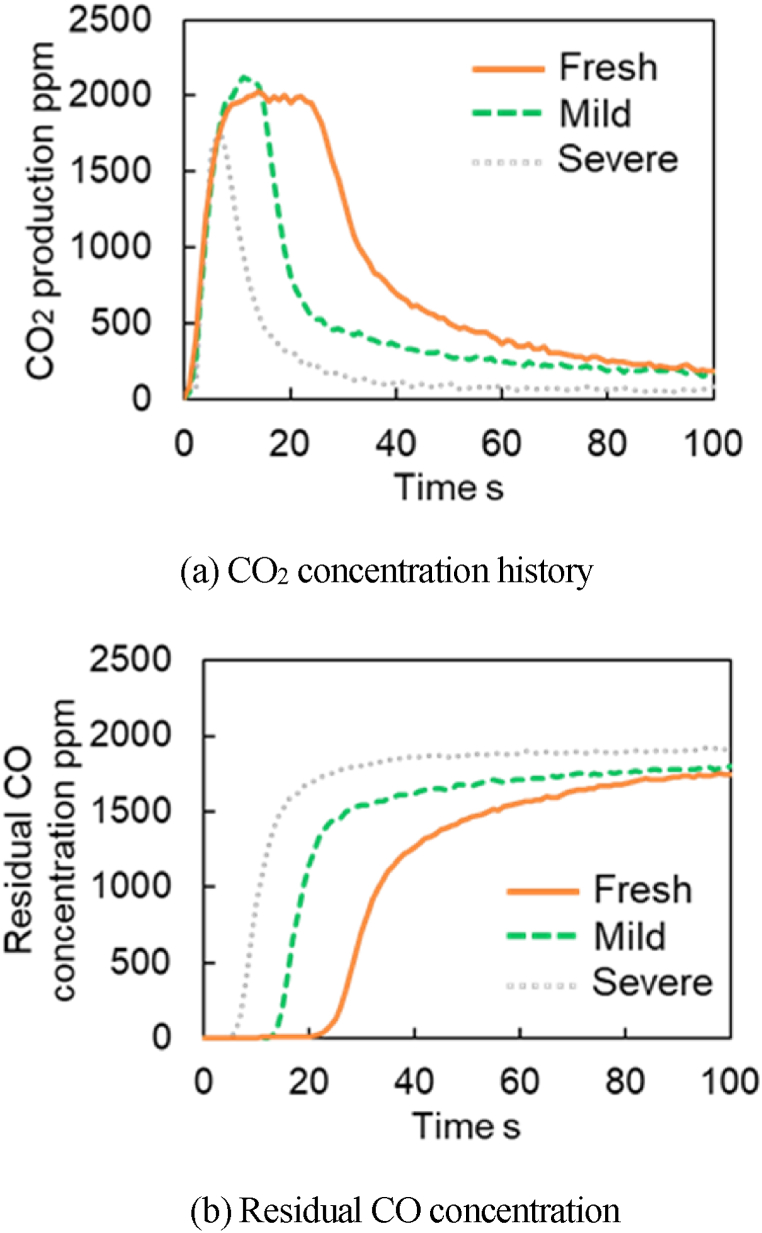
Experimental results of CO_2_ production test at 300 deg.C.

**Fig. 12:**
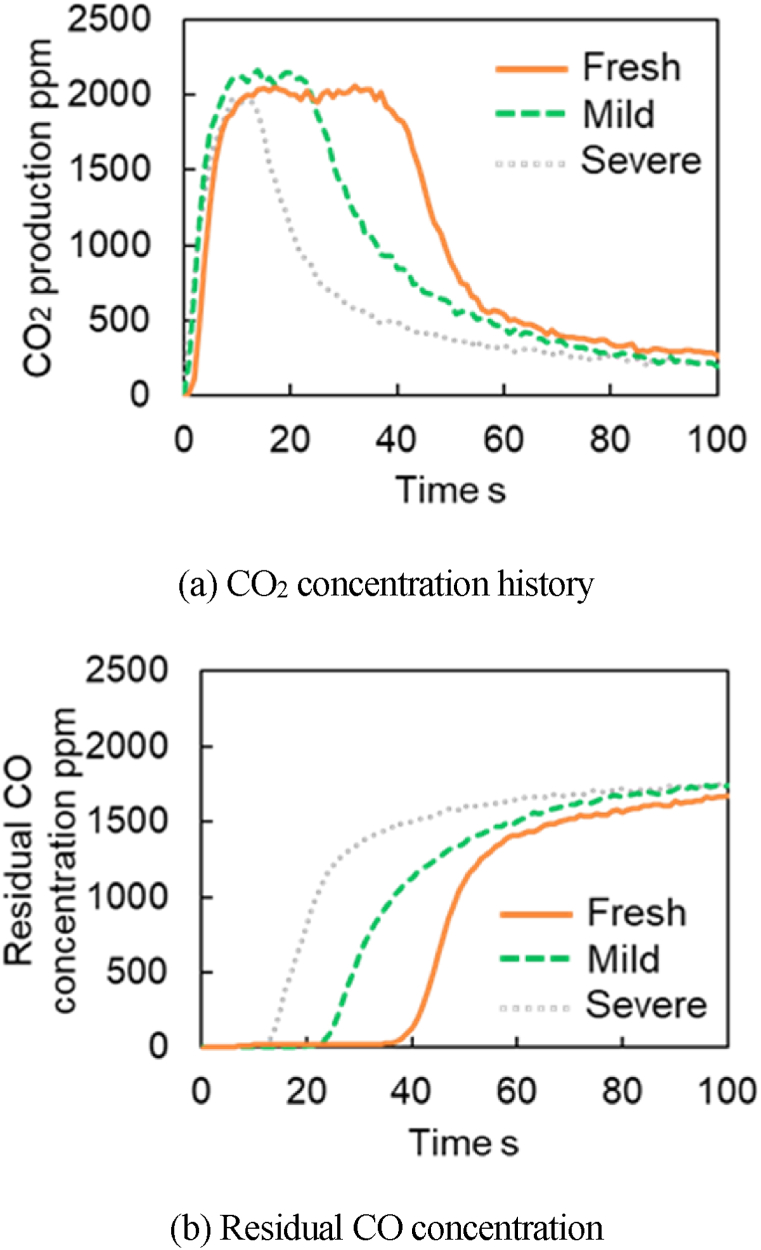
Experimental results of CO_2_ production test at 400 deg.C.

The experimental results of CO_2_ and residual CO concentrations for each catalyst at 300 °C are presented in Fig. 11a–b. These results show that the CO purification performances drop as the degree of degradation increases for both temperatures. The CO_2_ concentrations for all catalysts peak around 2000 ppm. Fig. 11 shows that the peak CO_2_ concentrations for Fresh CZ catalyst continue to produce for about ∼25 s, ∼15 s for mild catalyst, and <10 s for severe catalyst. Both figures show that the reaction rate of R29 decreases when catalyst aging increases or the degradation progress in which O atoms adsorbed from OSC move to the TPB. Therefore, the O atom supply to the TPB in degraded catalysts cannot be maintained, and then less CO_2_ is produced from R19 reactions. Fig. 12a–b report the CO_2_ and residual CO concentrations for each catalyst at 400 °C. The results show that the peak CO_2_ concentrations for 400 °C are more prolonged than that of 300 °C. As a result, the OSC adsorbs more O_2_. This is because, at higher temperatures(400 °C vs 300 °C), more O_2_ is adsorbed on S2, S3, and S4 considered in the model. In other words, this is because more oxygen atoms are adsorbed to each active site by the R17, R29, and R30 reactions when considered in the reaction scheme considered in the model.

## 5 Results and discussions

### 5.1 Estimation of activation energy and pre-exponential factor

**Fig. 13:**
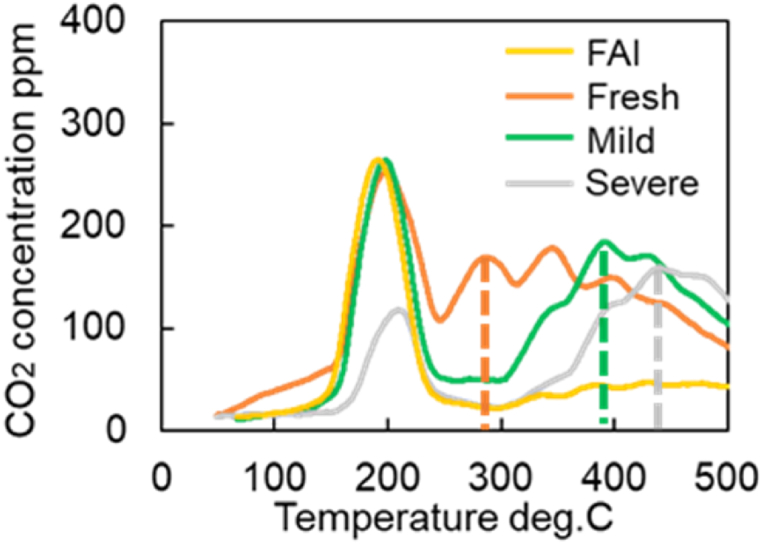
Results of moving average processing for CO-TPR test results at 20 deg.C/min (Dot line: second peak temperature for each catalysts).

**Fig. 14:**
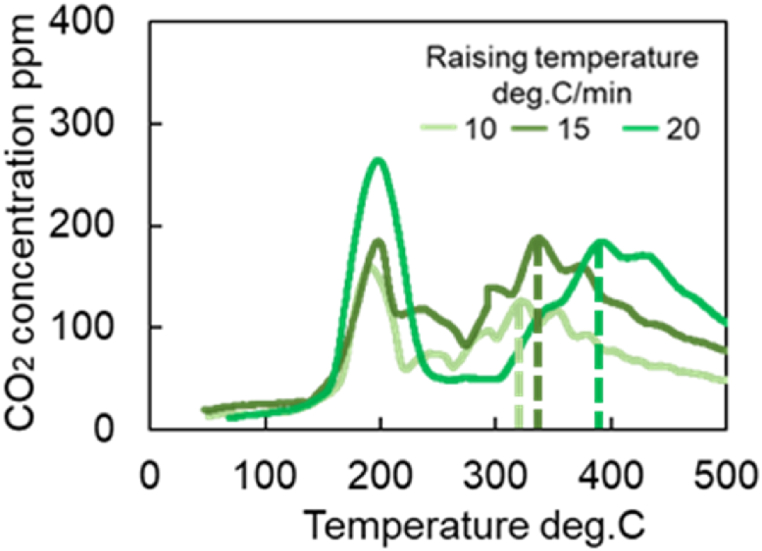
Results of moving average processing of CO-TPR test results for the Mild catalyst(Dot line: second peak temperature for each conditions of raising temperature).

It is necessary to estimate the temperature at which the second peak of CO_2_ production occurred in the CO-TPR test results (Fig. 10) to obtain the Ozawa plot for further analysis. The cause of the second peak in Fig. 9, Fig. 10a are challenging to determine due to the CO_2_ concentration oscillations. Therefore, the temperature is estimated using the moving average of 100 data points. Eq (9) represents the moving average formula for 100 data points in which $\bar{x_{i}}$ is data after moving-average processing and $x_{i}$ is the measured CO_2_ concentration per second. To clearly capture the peak values, a moving average process was performed as shown in Fig. 13. Fig. 13 shows the CO_2_ concentrations of all catalysts by using the moving average data from Fig. 9a. Fig. 14 represents the moving average results of the mild catalyst from Fig. 10a under different rising temperatures. When the rising temperature rate increase, fewer CO_2_ production oscillations are observed, then it is easy to estimate and extract the temperatures of the second peak of CO_2_ production, represented by the dashed lines in Fig. 13, Fig. 14. The more the catalyst degrades, the higher the temperature of the second peak.

(9) $$\begin{array}{c} \bar{x_{i}}=\frac{\sum_{i=1}^{100}x_{i}}{100} \end{array}$$

The following background suggests that Fast OSC contributes mainly to the second peak.

**Fig. 15:**
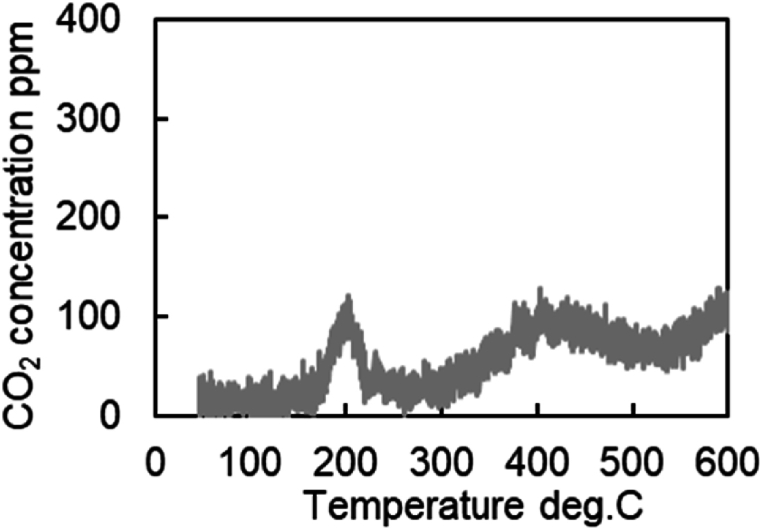
Results of CO_2_ concentration history of CO-TPR test at Severe catalyst and 10 deg.C/min.

**Fig. 16:**
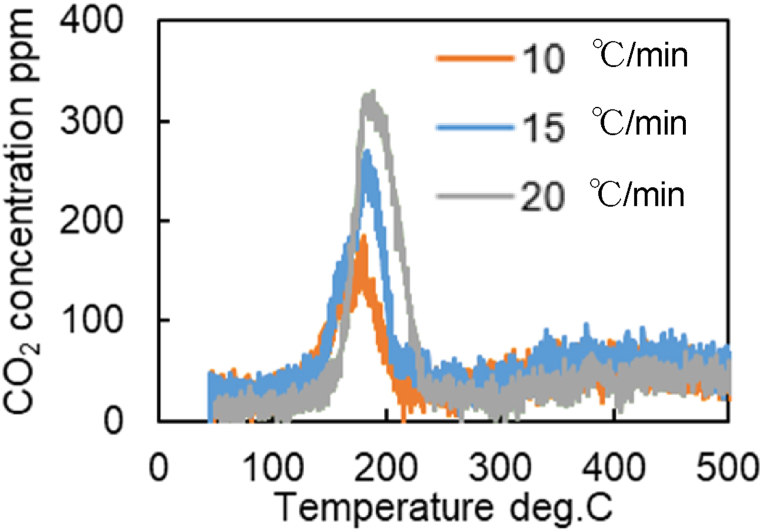
Results of CO_2_ concentration history of CO-TPR test at Fresh Al_2_O_3_ catalyst at each condition.

Fig. 15 shows the results of CO-TPR tests conducted on Severe catalysts at a temperature increase rate of 10 °C/min. The results show the presence of the first peak near 200 °C, the second peak near 400 °C, and the third peak after 600 °C. Thus, several peaks occur in the measured CO_2_ concentration when the temperature changes at a constant rate. In addition, as shown in Fig. 16, only one peak was affirmed in the Fresh Al_2_O_3_ catalyst results at all rates of temperature increase conditions. In other words, the presence of the CZ was found to cause the presence of the second and subsequent peaks. In addition, since OSC has both fast and slow reaction rates, OSC is separated into Fast OSC and Slow OSC for consideration in this study. A possible reason for the appearance of multiple peaks is that the multiple peaks appear due to the different reaction rates of each active site, suggesting that four active sites are required for this model.

**Fig. 17:**
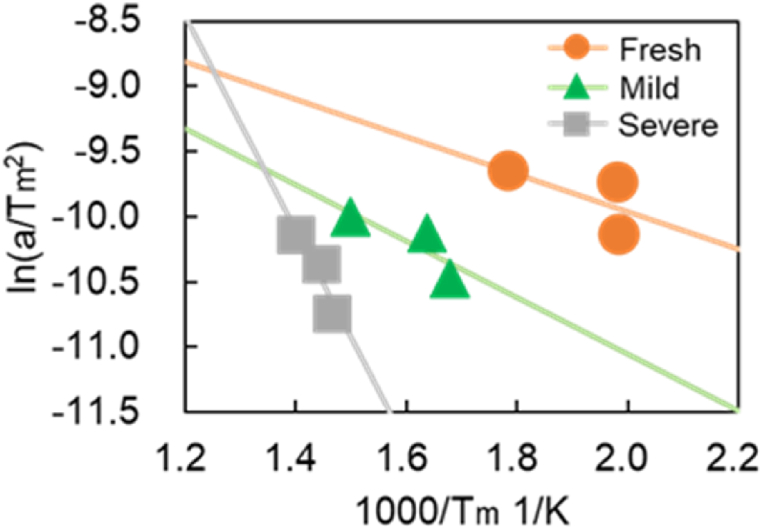
Results of analysis using an Ozawa plot for each catalyst (Fresh CZ, Mild, and Severe).

Next, an Ozawa plot is created using the peak temperature and increase rate for Fresh CZ, mild, and severe catalysts, as presented in Fig. 17. A linear approximation is performed using the least squares method for all catalysts to determine the activation energy and the pre-exponential factor. Table 12 lists the activation energy and the pre-exponential factor estimated from calculating the slope and the intercept term, as explained in the previous section. It is observed in Fig. 17 that the slope and the activation energy increase with increased catalyst aging degree. As shown in Fig. 10a, CO_2_ production slowly oscillates under a higher temperature raising rate. Consequently, small deviations from the approximate straight line are observed in Fig. 17. The slope of the severe catalyst varies significantly compared to that of Fresh CZ and Mild catalysts. It is possible that the severe catalyst endurance treatment temperature of 1000 °C caused the phase separation of the OSCs. In Fig. 17, the results for the Fresh CZ catalyst are difficult to draw a straight line, but as mentioned earlier, the second and subsequent peaks are found to be CZ-derived peaks from Fig. 9, Fig. 16. Therefore, in order to improve the prediction of the purification performance of Fresh CZ catalysts in the future, the following will be considered. On the experimental side, the test will be conducted under more temperature increase rate conditions, especially in Fresh CZ catalysts. On the experimental analysis side, we will seek a method of defining peaks that is different from that of degraded catalysts. With respect to model improvement, new active sites will be considered.

**Table 12:** Calculation of activation energy in R29 backward reaction for each catalyst.

Catalyst	Activation energy E kJ/mol	Pre-exponential factor A
Fresh CZ	11.94	1.198
Mild	17.98	2.589
Severe	68.14	3.250 × 10^4^

### 5.2 Estimation for active site density of OSC

**Fig. 18:**
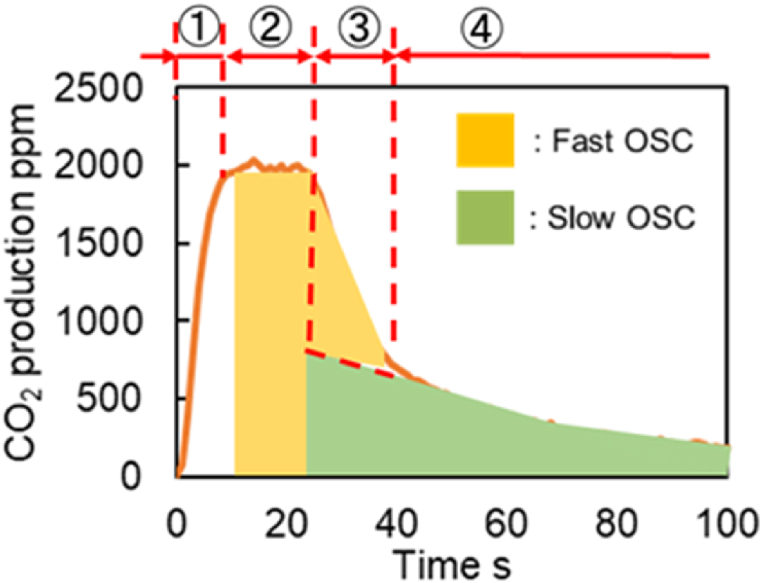
Boundaries at each active site in CO_2_ production test for Fresh catalyst.

The OSC active site density in each catalyst is calculated based on the CO_2_ production tests in which its reactions are depicted and previously explained in Fig. 4. Fig. 18 shows CO_2_ production for stages 1–4 for Fast OSC and Slow OSC. At stage 1, the reactions in PGM and TPB cause a rapid CO_2_ to increase at the start of the reaction. In stage 2, the oxygen storage in the precious metal is consumed; however, the oxygen storage in the Fast OSC can help maintain good purification performance. Then, the oxygen storage in the Fast OSC is depleted in stage 3, and the CO_2_ production reaches its peak state, and there is a switch to the oxygen storage in the Slow OSC. In the final stage 4, the oxygen adsorption in the slow OSC decreases, while the CO_2_ production generally decreases at a constant rate. Based on these reaction stages, the active site densities of Fast OSC and Slow OSC can be calculated using the Fresh CZ catalyst, as shown in Fig. 18. The boundary between Fast OSC and Slow OSC is defined in stage 3. The slope defines this boundary after the inflection point at which the decline rage in the purification performance is slower using the least-square method. The integrated values and density of each active site are estimated in addition to the boundaries. Table 13 lists the values estimated by the CO_2_ production tests. These values show that the active site densities of Fast OSC (S3) and Slow OSC (S4) decrease with catalyst aging or degradation. The background of this calculation is as follows.

**Table 13:** Active site density for Fresh CZ, Mild, and Severe catalysts.

	Fast OSC σ(S3) mol/m^3^	Slow OSC σ(S4) mol/m^3^
Fresh CZ	19.8	35
Mild	14.9	20
Severe	6.9	10

**Fig. 19:**
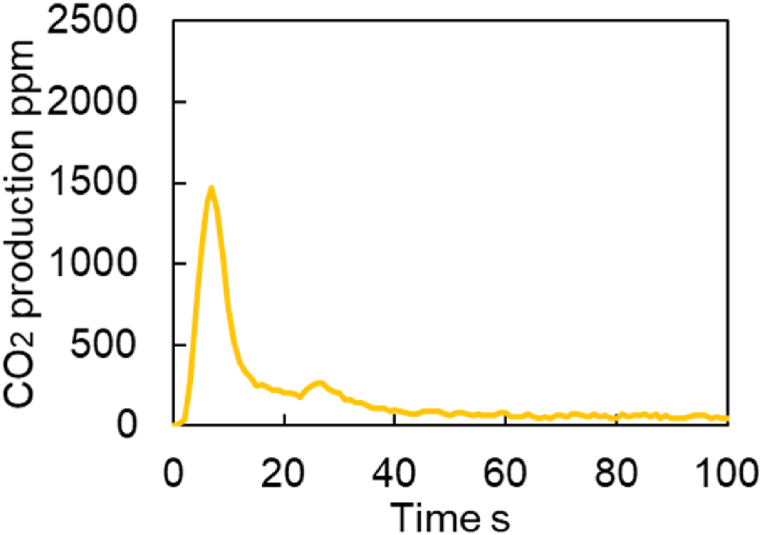
Experimental results of CO_2_ production test for Fresh Al_2_O_3_ catalyst at 300 deg.C.

Based on the experimental results of the catalyst with Pd on Al_2_O_3_, the assumption from step 1 to step 4 seems reasonable. Fig. 19 shows the results of a CO_2_ production test carried out at 300 °C on a Fresh Al_2_O_3_ catalyst. The test conditions are shown in Table 14. The amount of CO_2_ produced increased with the start of the reaction, peaking at around 1500 ppm and peaking out soon afterward.

**Table 14:** Experiment conditions for the CO_2_ production test.

CO	ppm	2000
O_2_	ppm	1000
N_2_	%	Balance
Space velocity	h^−1^	7.6 × 10^4^
Temperature	deg．C	300
Test time	s	15000

**Fig. 20:**
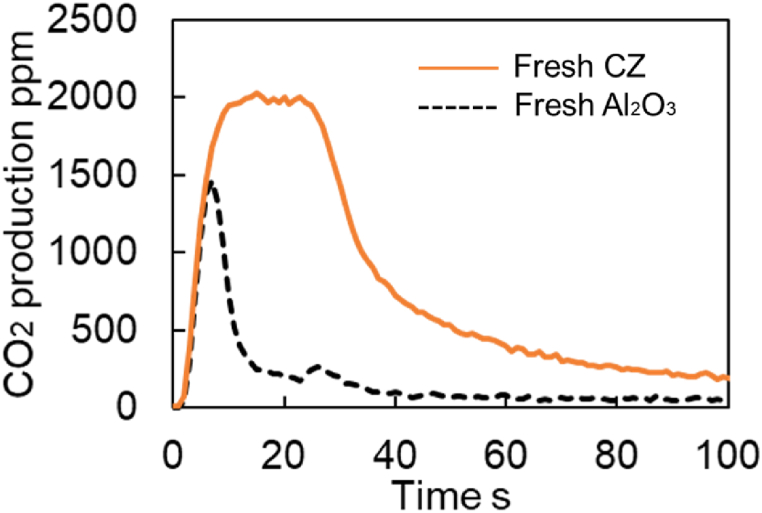
Experimental results of CO_2_ production test for Fresh Al_2_O_3_ and Fresh CZ catalysts at 300 deg.C.

A comparison of the results for Fresh Al_2_O_3_ and fresh CZ catalysts is shown in Fig. 20. From this comparison, it was found that Steps (1) to (4) are assumed in this study, shown in Fig. 18.

First, in step ①, CO_2_ histories of both catalysts have the same slope. Therefore, we thought that the reaction proceeds mainly on the precious metal in step ①. CO_2_ peak value was smaller for the Fresh Al_2_O_3_ catalyst than for the Fresh CZ catalyst, declining to about 300 ppm during step ①. This is because the Fresh Al_2_O_3_ catalyst absorbs less O_2_ and the peak value of CO_2_ production is reduced. However, since the slope of CO_2_ production is the same, the reaction can be judged to be on precious metal in step ①.

Second, in step ②, the CO_2_ history gradually decreases while fluctuating between 100 and 200 ppm for Fresh Al_2_O_3_ catalyst. On the other hand, the CO_2_ history maintains 2000 ppm for Fresh CZ catalyst. For these reasons, in step ②, it was determined that CO_2_ is produced by oxygen from the Fast OSC, which has a fast reaction rate.

Third, in step ③, the CO_2_ history gradually decreases while fluctuating between 100 and 200 ppm for Fresh Al_2_O_3_ catalyst as the same as in step ②. On the other hand, the results for the Fresh CZ catalyst showed a rapid decrease in CO_2_ history from 2000 ppm. In step ③, we think that this Fast OSC-derived O_2_ gradually decreased.

Finally, in step ④ of following 40 s at starting measurement, the CO_2_ concentration is generally equal to 0 in the result for the Fresh Al_2_O_3_ catalyst, and the slope of the concentration change is close to 0. In contrast, following 40 s at starting measurement, the Fresh CZ catalyst results show a gradual decrease from 500 ppm and a slower rate of CO_2_ production. Judging from this, we considered that step ④ is largely related to Slow OSC. However, since it is difficult to assume that Slow OSC was suddenly involved from step ④, the slope and intercept after step ④ were determined by the least-squares method, and the contribution of Slow OSC was assumed to be present from the start of step ③. Since the above background was obtained for the Fresh CZ catalyst, the analysis for the degraded catalyst was performed assuming steps ① through ④ as for the Fresh CZ catalyst.

### 5.3 Validation of activation energies, pre-exponential factors, and OSC active site density

A 1D TWC model was developed using Exothermia Suite software to validate the activation energies, pre-exponential factors, and OSC active site density. The extended model from Ref. [11] was also utilized to predict the TWC purification performance degradation due to catalyst aging. The previous model developed by the authors [11] did not account for the wash-coat diffusion and ignored the concentration of each chemical species using the 0D intra-layer mesh model. Also, the prior model assumed that the inlet conditions and wash-coat distributions were uniform, and the simulations were carried out only for Fresh CZ and Mild catalysts. These assumptions and shortcomings are addressed in this work by treating the PGM (S1), TPB (S2), Fast OSC (S3), and Slow OSC (S4) shown in Fig. 1. Table 4 lists the active site density for each catalyst for the modeling, while the inlet gas compositions and temperature evolution are initialized using measured values.

**Fig. 21:**
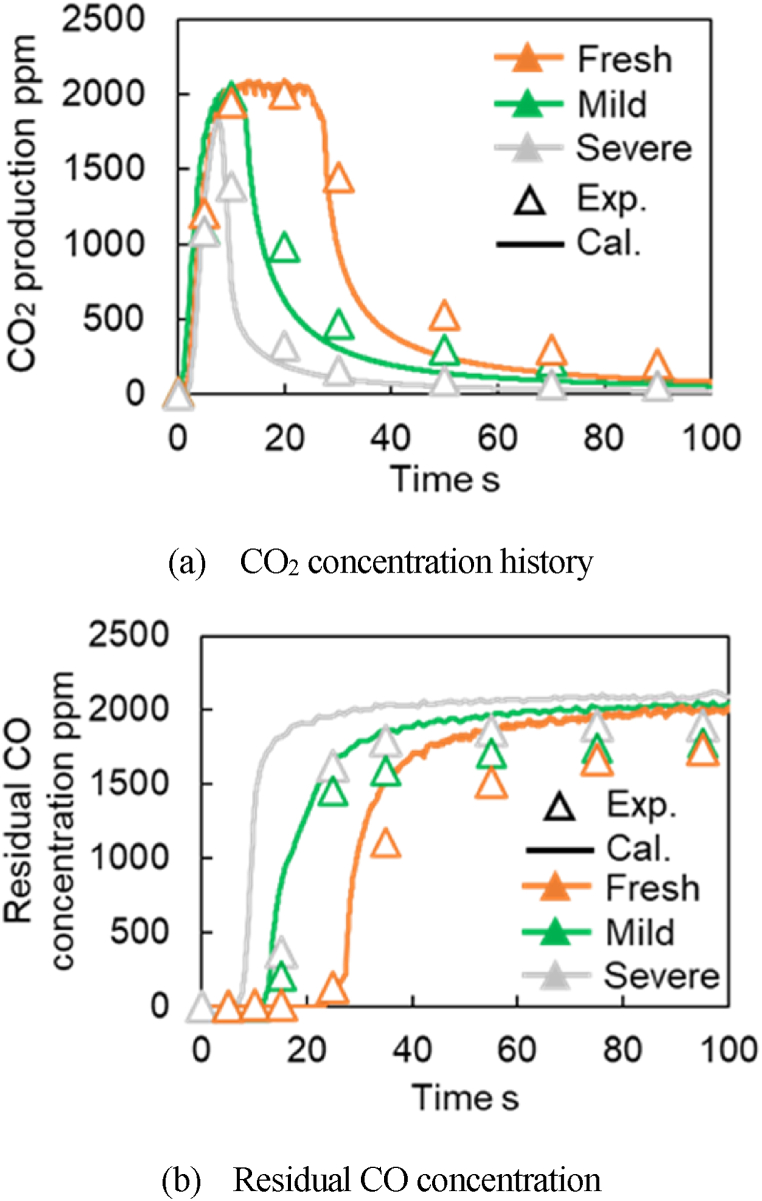
Verification of CO_2_ concentration and the residual CO concentration in the CO_2_ production test (symbol: Experimental value, Line: Calculated value).

**Fig. 22:**
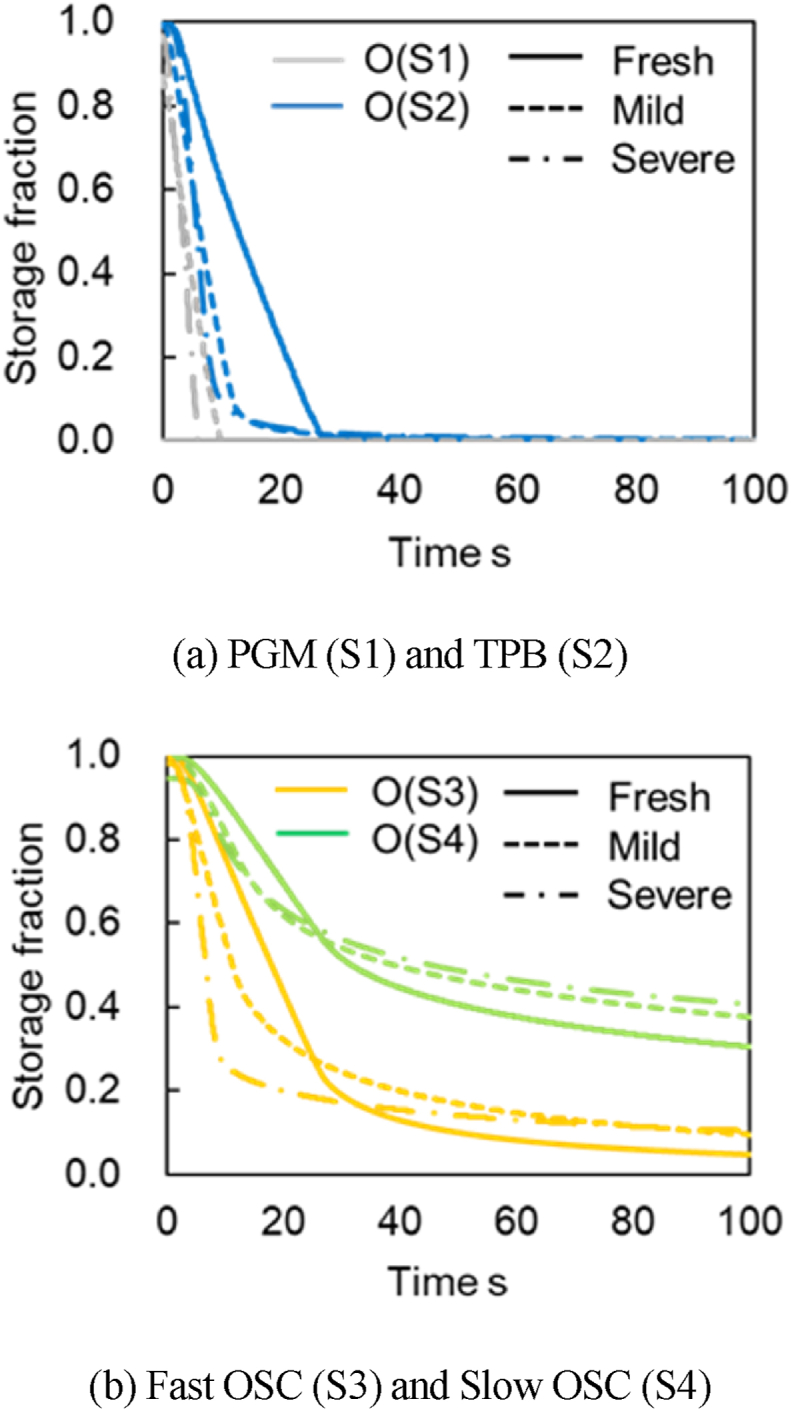
Verification of Storage fraction in CO_2_ production test.

Fig. 21a–b presents good validations of the CO_2_ concentration and the residual CO concentration for Fresh CZ, Mild, and Severe catalysts. Fig. 21a–b shows that the predicted CO_2_ productions have similar tendencies when they reach the maximum concentration at around 2000 ppm and continue to produce at the same concentration for about 25 s for Fresh CZ, 10 s for mild, and <10 s for severe catalysts, respectively. These tendencies of the predicted results are the same as measured values. Slight predicted deviations from measured values are likely because the slow OSC is not included. Fig. 22 presents the predicted oxygen surface species coverage ratio at each active site. The coverage ratio decreases with the O_2_ adsorption amount. At all active sites, the reduction rate in the coverage ratio from the start of the reaction is more significant when the aging or degradation degree increases. For PGM (S1) shown in a gray line in Fig. 22a, the fastest time is zero because it is the most active site where the reaction occurs in a degraded catalyst. In TPB (S2) shown in a blue line, the coverage ratio becomes zero earlier as the catalyst aging increases. In Fast OSC (S3), represented by an orange line in Fig. 22b, it can be seen that the oxygen adsorption species at PGM (S1) and TPB (S2) are reduced, and that of S3 decreases for all catalysts. In Slow OSC (S4) represented by a green line in Fig. 22b, the decreasing time and rate are the slowest in all active sites. The reason is likely because oxygen is provided from Fast OSC (S3) to TPB (S2) through the reverse reaction of R29. The conversion reaction R19 occurs from CO to CO_2_ on S2. Moreover, the reverse reaction in R8 is assumed to be caused by a slow oxygen movement from Slow OSC (S4) to Fast OSC (S3).

**Fig. 23:**
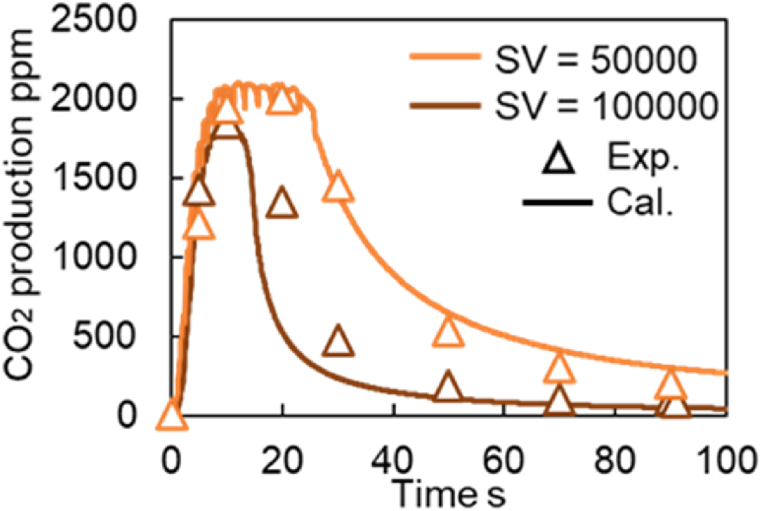
Verification of CO_2_ concentration in CO_2_ production test under different SV conditions at Fresh CZ catalyst.

**Fig. 24:**
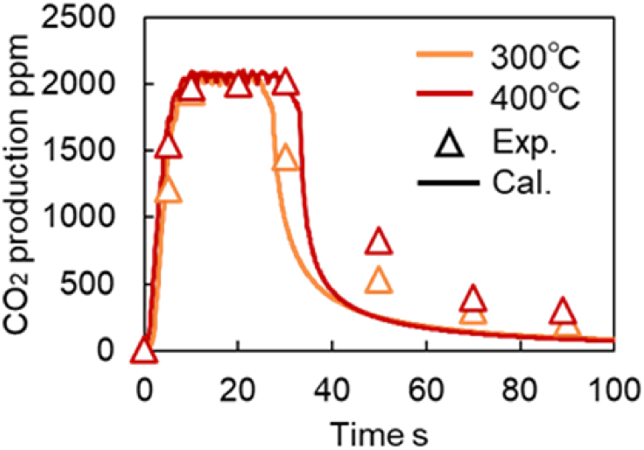
Verification of CO_2_ concentration in CO_2_ production test under different temperature conditions at Fresh CZ catalyst.

To further evaluate the versatility of the model, CO_2_ production tests were conducted using Fresh CZ catalysts under multiple space velocity (SV) and temperature conditions. Fig. 23 shows the validations of CO_2_ concentrations in the CO_2_ production test under two space velocities (50,000 and 100,000), while the validation results at 300 and 400 °C are shown in Fig. 24. It is observed in Fig. 24 that the predicted values deviate from the measured ones after 40-s. This is because this model did not adequately represent the temperature dependence of the Slow OSC. Since this study focuses on automotive TWC applications and the oxygen subject to perturbation is primarily the Fast OSC, the exact values of the reaction rate parameters A and E for the Slow OSC were not calculated. Therefore, Slow OSC does not accurately reproduce the temperature dependence. Overall, the validation results in Fig. 23, Fig. 24 can reproduce actual phenomena. In other words, the TWC model constructed in this study can accurately predict purification performance under various temperatures and space velocities.

## 6 Conclusions

This work investigates the effects of qualitative factors of OSC under thermal degradation on the TWC purification performance using experimental and 1D modeling analysis. The degradation state and accurate TWC model can be predicted and developed based on OSC. Purification performances of the TWC model under various catalyst aging degrees can be predicted with reasonable accuracy. Summaries are given as follows.

(1) From the CO-TPR test, changes in OSC characteristics with aged catalysts are confirmed at each rate of temperature increase. The Ozawa plot results show that the increased activation energy of the reaction at OSC is confirmed for aged catalysts. An accurate OSC model is constructed using the activation energy estimated from the analysis results.
(2) A small temperature oscillation can also cause CO_2_ concentration oscillations. Less CO_2_ production oscillations are reduced at faster temperature rate.
(3) CO purification performance decreases when the degradation degree increases for all investigated temperatures (300–400 °C). As the degradation progresses under higher degrees of catalyst aging, a slower reaction rate R29 occurs, in which O atoms adsorbed from OSC move to TPB. Therefore, the supply of O atoms to TPB in the degraded catalysts cannot be maintained, so CO_2_ production is minimal in R19 reactions. The proposed reaction steps are feasible under multiple experimental conditions, space velocities, and temperatures. The relationship between thermal degradation and CO oxidation can be predicted adequately.

The catalyst degradation and CO oxidation in the CO_2_ production test should also be investigated under high-temperature ranges amid the capability of the experimental device. Further prediction accuracy improvement of the OSC model should be considered in future works by formulating the active site density and activation energy changes with degradation degree. The CO-TPR tests show that higher temperature increase rates should be further performed to avoid high oscillations, and then good linearities of Ozawa plot results can be used to improve the activation energy predictions for the oxygen release reactions in Fast OSC. After the model's refinement with OSC, the model's validity should be investigated for other chemical species.

## CRediT authorship contribution statement

**Sota Aoyama:** Writing – original draft, Investigation, Formal analysis. **Yunosuke Kubo:** Investigation. **Ratnak Sok:** Writing – review & editing, Supervision, Conceptualization. **Jin Kusaka:** Writing – review & editing, Supervision, Project administration, Funding acquisition, Conceptualization.

## Declaration of competing interest

The authors declare that they have no known competing financial interests or personal relationships that could have appeared to influence the work reported in this paper.
